# An assessment of the interaction for three *Chrysanthemum indicum* flavonoids and α‐amylase by surface plasmon resonance

**DOI:** 10.1002/fsn3.1349

**Published:** 2019-12-16

**Authors:** Pao Li, Zhao Huang, Yin She, Si Qin, Wanru Gao, Yanan Cao, Xia Liu

**Affiliations:** ^1^ College of Food Science and Technology Hunan Provincial Key Laboratory of Food Science and Biotechnology Hunan Agricultural University Changsha China; ^2^ Hunan Co‐Innovation Center for Utilization of Botanical Functional Ingredients Changsha China

**Keywords:** *Chrysanthemum indicum*, flavonoids, interaction, surface plasmon resonance, α‐amylase

## Abstract

This study evaluated the interaction of *Chrysanthemum indicum* (CI) flavonoids (luteolin, acacetin, and buddleoside) with α‐amylase. Surface plasmon resonance (SPR) assay showed their equilibrium dissociation constants (*K_D_*) are 1.9695 ± 0.12, 2.9240 ± 0.20, and 3.2966 ± 0.08 mM at pH 6.0, respectively. Furthermore, their binding affinities were influenced by KCl, MgCl_2_, and CaCl_2_. Enzymatic kinetic studies revealed that three flavonoids exhibited noncompetitive α‐amylase inhibitory activity. The inhibitory sequence is luteolin > acacetin > buddleoside, which was in accordance with the results of binding affinity from SPR. 1,1‐diphenyl‐2‐picryl hydrazyl radical assay demonstrated that antioxidant activities of three flavonoids were inhibited significantly with α‐amylase. Meanwhile, the study reveals that hydroxyl on C′‐4, C′‐5, and C‐7 of flavonoids play an important role on the interaction of three flavonoids with α‐amylase. Also, SPR could be used as sensor for rapid screening inhibitors of α‐amylase and provide useful information for the application of *C. indicum* flavonoids in food and pharmaceutical area.

## Graphical abstract

In this work, the kinetic constants, structure‐activity relationship, and influence factors of flavonoids and α‐amylase interaction were revealed. α‐Amylase activity and flavonoids antioxidant abilities were examined after interaction of flavonoids with α‐amylase.

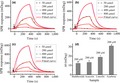

## 1 INTRODUCTION

Diabetes mellitus (DM), a metabolic syndrome characterized by high blood glucose, has been linked to multiple organ damage and dysfunction. At present, it has become the most prevalent chronic disease in the world (Guariguata et al., 2014). On 6 March 2016, the world health organization released global diabetes report for the first time. The number of patients suffering from the disease was triple increased since 1980, and this number was predicted to rise to 642 million by 2040 (Cho, 2016). Type 2 diabetes (T2D) accounts for more than 90% of all cases of diabetes globally. In recent years, strong relationship between the postprandial hyperglycemia and T2D has been demonstrated (Ceriello et al., 2008, 2004). Therefore, normalizing blood glucose level is very important in the prevention and treatment of T2D. Many studies have indicated that postprandial glucose levels can be regulated through α‐amylase inhibition (Lee et al., 2012; Park, Lee, & Han, 2017).

Acarbose, commercial available α‐amylase inhibitor, is typical therapeutic agent used to control postprandial glucose concentration. However, it has been reported to cause some gastrointestinal side effects, such as diarrhea, flatulence, and abdominal pain (Shah, Khalil, Ul‐Haq, & Panichayupakaranant, 2017; Yang, He, & Lu, 2014). Compared with the synthetic drugs, the natural molecules from plant have become a more acceptable alternative for treating T2D. Flavonoids are a class of natural small molecules with broad biological activity (Shen, Xu, & Lu, 2012; Tomás‐Barberán & Andrés‐Lacueva, 2012). Recently, they have received much attention for their inhibitory activity against α‐amylase and relatively low toxicity to animals (Cao & Chen, 2012; Lu et al., 2017). Further, a series of studies have demonstrated that the structure and concentration of flavonoids and structure of α‐amylase may greatly influence the extent of the flavonoids/α‐amylase interaction (Cao & Chen, 2012; Lo et al., 2008; Wang, Du, & Song, 2010). So, flavonoids have been considered as a good source for screening of α‐amylase inhibitor.

*Chrysanthemum indicum* (CI) is a kind of herbaceous plant. Its flowers have been used for several centuries as a traditional Chinese medicine to treat various infectious diseases, immune‐related disorders, and eye diseases (Cheng, Li, & Hu, 2005; Zhu, Yang, Yang, Yang, & Zhou, 2005). Flavonoids are important bioactive components in the flowers of CI, including buddleoside, acacetin, and luteolin (Wang et al., 2000). The content of these flavonoids compounds has been used as the quality standard of CI. To the best of our knowledge, the interaction between CI flavonoids and α‐amylase has not been clearly demonstrated in detail.

**Figure 1:**
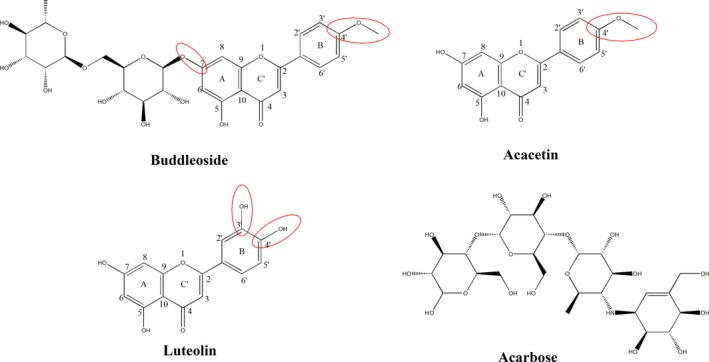
Chemical structures of buddleoside, acacetin, luteolin, and acarbose

Surface plasmon resonance (SPR) is considered one of the most powerful techniques for evaluating the affinity kinetics of molecular interaction, which allow accurate estimation of distinct association/dissociation rate constants and equilibrium parameters in different reaction models without labels (Tan et al., 2014; Tiwari et al., 2014). In this study, the binding kinetics of CI flavonoids (buddleoside, acacetin, and luteolin) and α‐amylase were monitored in vitro, and the effects of the external factor on their binding affinities were also analyzed using SPR biosensor. On this basis, the inhibitions of three flavonoids on α‐amylase activity were examined, and a reasonable inhibiting mode was proposed. Furthermore, we studied whether the antioxidant activity of these active constituents can be affected during the interaction with α‐amylase by 1,1‐diphenyl‐2‐picryl hydrazyl (DPPH) radical assay. The difference of the interaction between the three flavonoids and α‐amylase was analyzed based on the molecular structures of three flavonoids (Figure 1). The obtained results may be able to provide useful information for the more effective application of CI in food and pharmaceutical area.

## 2 MATERIALS AND METHODS

### 2.1 Apparatus

A commercial BI‐2000 SPR instrument (Biosensing Instrument Inc.) was used for all SPR experiments in this study. The bare Au sensor chip was obtained from Biosensing Instrument Inc. The preparation of Au sensor chip can be referred to our previous published paper (Liu et al., 2014). A flow delivery system incorporated in the BI‐SPR platform pumped samples onto the SPR sensor chip at a flow rate of 10 μl/min. The 0.01 M PBS (pH = 6.0) buffer was used as the running buffer. The BI‐SPR 2000 control software (version 2.2.0.) was used to perform instrument operation and data processing. The Varioskan Flash (Multiskan GO 1510, Thermo Fisher Scientific) was used for the α‐amylase inhibitory activity and DPPH radical assays.

### 2.2 Reagents

Buddleoside (purity: 99.37%), acacetin (purity: 99.8%), and luteolin (purity: 98.92%) were purchased from Chengdu Manst Biotechnology Co. Ltd. *Bacillus subtilis* α‐amylase was purchased from Shanghai Ryon Biological Technology Co. Ltd.. DPPH and soluble starch were purchased from Changsha LongHe chemical and glass experimental materials limited Co. Ltd. Acarbose (purity ≥ 98%), 3‐mercaptopropionic acid (MPA), N‐hydroxysuccinimide (NHS), and 1‐ethyl‐3‐(3‐dimethylaminopropyl) carbodiimide hydrochloride (EDC) were purchased from Sigma‐Aldrich. All reagents were of analytical grade and used without further purification. The ultrapure water was used throughout this work.

### 2.3 SPR measurement of three flavonoids and α‐amylase interactions

Binding assay of three flavonoids to α‐amylase was carried out using the SPR sensor. The immobilization of α‐amylase on the chip surface was performed using a standard amine coupling procedure as described previously (Liu, Luo, Li, She, & Gao, 2017). The acceptable immobilization level of the α‐amylase (referred to as bound and final α‐amylase responses) was about 300 mDeg. After the stable baseline was obtained, different concentrations of flavonoids (50–800 μM) were injected over the chip surface coated with α‐amylase, respectively. The SPR angle was monitored until the baseline stabilization. To enable reuse of the SPR chip, the chip surface could be regenerated using 2 mM NaOH after each measurement. Regeneration parameters were based on the strength of interaction between the analyte and α‐amylase. The chip surface was rinsed by PBS between each step. All the experiments were repeated three times, and kinetic parameters (*k_a_*, *k_d_*) were deduced by nonlinear fitting of the primary sensorgram data based on the 1:1 Langmuir‐binding model using the BI‐SPR 2000 control software (version 2.2.0.). The model has been widely used in protein–ligand binding analysis and can be calculated through the following formula (Gombau et al., 2019; Islam, Shen, Gurgel, Rojas, & Carbonell, 2014; Lee, Jeong, Jones, & Kim, 2011):

(1) $$\frac{\mathrm{dR}}{\mathrm{dt}}=k_{a}C\left(R_{\max}-R\right)-k_{d}R$$

where *R* is the SPR signal at time *t*, and *C* is the concentration of the analyte. *R*_max_ is the maximum analyte binding capacity in SPR signal. *k_a_* is the association rate constant and *k_d_* is the dissociation rate constant.

### 2.4 Effect of pH and salt on the interaction between three flavonoids and α‐amylase

The effect of pH on the interaction between three flavonoids and α‐amylase was carried out within the pH range (3–9) based on the method described in the above experiment. As is known to all, metal ions play a crucial role in maintaining normal physiological function of the α‐amylase. Moreover, salt is also widely used in food industry. To evaluate whether KCl, MgCl_2_, and CaCl_2_ can interfere with the interaction between flavonoids and α‐amylase, a series of 200 μM flavonoids with a various concentrations of KCl (0.02–0.3 M), MgCl_2_ (0.02–0.25 M), or CaCl_2_ (0.04–0.2 M) solutions were flowed over the chip surface modified with α‐amylase, respectively.

### 2.5 Effect of three flavonoids on α‐amylase activity

The changes of α‐amylase activity after adding different concentrations of the three flavonoids were investigated according to previously reported method with a slight modification (Zengin, 2016). In brief, 0.05 ml α‐amylase (300 mM in PBS buffer, pH = 6.0) was incubated with 0.5 ml of each of the three flavonoids at various concentrations (20, 40 and 80 μM) for 10 min at 37°C, respectively. Then, 2 ml of starch solution (0.1 M in PBS buffer, pH = 6.0) was added to the above mixture. After incubation for 10 min at 37°C, 0.5 ml of 0.01 M iodine‐potassium iodide solution was added to start the reaction. Finally, PBS (pH = 6.0) was added to give a final volume of 8 ml. Thereafter, the assay was carried out by measuring the absorbance at 560 nm using the Microplate Spectrophotometer. All experiments were performed in triplicates, and the inhibitory percentage of α‐amylase activity was calculated through the following formula (Shah et al., 2017):

(2) $$\text{Inhibition}\;\text{ratio}\;\left(\%\right)=\left(1-\frac{A}{A_{0}}\right)\;\times 100\%$$

where *A*_0_ is the absorbance without flavonoids, and *A* is the absorbance with flavonoids.

To further explore the inhibitory type of three flavonoids on α‐amylase, kinetic analysis was carried out by using Lineweaver–Burk plots. Starch was used as substrate, and the inhibition kinetics of α‐amylase was evaluated by varying four different concentrations (0.25, 0.50, 1.00, and 1.25 mg/ml) of starch in the absence or presence of three flavonoids. The Lineweaver–Burk plots of the three flavonoids can be obtained from the double‐reciprocal plots between 1/[*S*] (starch concentration) and 1/[*V*] (reaction rate). Besides, different concentrations of flavonoids (0, 5, and 10 μM) were used and the three Lineweaver––Burk plots for each flavonoid can be obtained. The *K_i_* (inhibition constant) value was obtained from the least‐squares regression line of the slopes of Lineweaver–Burk plots versus the corresponding flavonoid concentrations [*I*] (Kandra, Gyémánt, Zajácz, & Batta, 2004; Yang et al., 2014). The formula is

(3) $$\text{slope}=\frac{K_{m}}{V_{\max}}\left(1+\frac{\left[I\right]}{K_{i}}\right)$$

*K_i_* = intercept of Equation (3) on *x* axis (Yang et al., 2014)

### 2.6 Effect of α‐amylase on antioxidant activity of three flavonoids

The DPPH assay was performed to assess effect of α‐amylase on antioxidant activity of three flavonoids as following procedure (Kim et al., 2014). 2 mM DPPH solution was prepared by dissolving 0.0787 g of DPPH in 100 ml of anhydrous ethanol and stored at −20°C. α‐Amylase (300 μM) and three flavonoids (2.5–125 μM) were prepared with PBS (pH = 6.0) and ethanol, respectively. Samples were prepared by mixing flavonoids solution (without or with 10 μl α‐amylase), 100 μl of DPPH, and anhydrous ethanol. Then, samples were incubated for 10 min at room temperature. Absorbance at 517 nm was measured in the spectrophotometer. DPPH radical scavenging activity was calculated as follows (Liu et al., 2017):

(4) $$\text{DPPH}\;\text{radical}\;\text{scavenging}\;\text{activity}\;\left(\%\right)=\left(1-\frac{A_{i}-A_{j}}{A_{c}}\right)\times 100\%$$

in which *A_C_* is the absorbance of the DPPH (100 μl DPPH and 100 μl ethanol), *A_i_* is the absorbance of the DPPH and sample (100 μl DPPH, 50 μl flavonoids, and 50 μl ethanol, or 100 μl DPPH, 50 μl flavonoids, 10 μl α‐amylase, and 40 μl ethano1), and *A_j_* is the absorbance of the blank sample (50 μl flavonoids and 150 μl ethanol). All experiments were carried out in triplicate, and the results were expressed as mean ± RSD. IC_50_ values were obtained based on plotting the percentage of DPPH radical scavenging activity against the flavonoids concentration.

### 2.7 Statistical analysis

The data were expressed as means ± relative standard deviation (*n* = 3). Statistical analysis was compared using a one‐way analysis of variance in SPSS 18.0 (SPSS, Chicago, IL, USA), with *p* < .05 being considered statistically significant.

## 3 RESULTS AND DISCUSSION

### 3.1 Interaction of three flavonoids and α‐amylase

**Figure 2:**
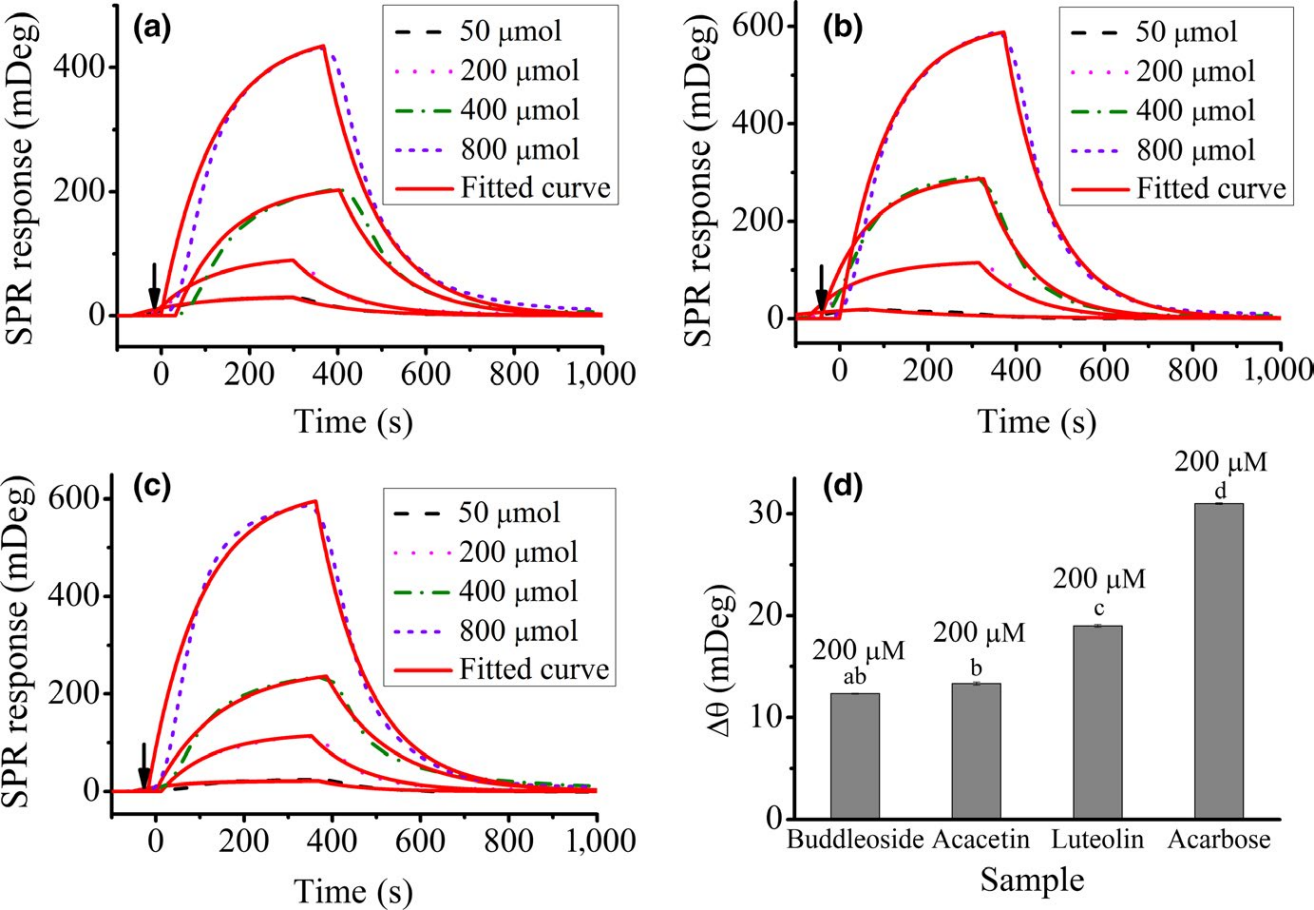
SPR kinetic curves of binding process between buddleoside (a), acacetin (b), luteolin (c), and immobilized α‐amylase. The black arrows indicate the time point at which ligands are added. (d) SPR responses respect to three flavonoids (200 μM), and acarbose (200 μM) flowed over the α‐amylase‐modified chip surface, respectively. Results are expressed as the mean ± RSD (*n* = 3). Mean values followed by different letters are significantly different (*p* < .05)

The SPR sensorgrams in Figure 2 demonstrate the interaction of three flavonoids with α‐amylase immobilized on the chip surface. Acarbose is a commercial available α‐amylase inhibitor and can be used to control postprandial glucose concentration. In order to understand whether such an interaction is related to the flavonoid molecular structure, acarbose was used as positive control. Periodically throughout each experiment, the PBS (pH = 6.0) buffer was injected into the flow cell to serve as a baseline reference for removing any systematic drift over time, and the level of the three flavonoids and acarbose binding to the immobilized α‐amylase was measured based on the change of the SPR response. The SPR response obtained in each individual reaction cycle was recorded as a sensorgram, which is a real‐time pattern plotted as SPR response versus time (in seconds). Figure 2a–c is the SPR kinetic curves of binding process between buddleoside (A), acacetin (B), luteolin (C), and immobilized α‐amylase. It can be seen from the Figure 2a–c that the interaction of three flavonoids and α‐amylase is very obvious. The interaction kinetics can be subdivided in three distinct phases: association, steady state, and dissociation. The association and the dissociation of flavonoids and α‐amylase can be monitored with the increase and decrease in SPR response. Besides, the effective SPR response increased in proportion with the concentration of the flavonoids. The SPR curve was fitted based on the theoretical 1:1 model for calculating the association rate constant (*k_a_*), the dissociation rate constant (*k_d_*), and the equilibrium dissociation constant (*K_D_*). These kinetic parameters are given in Table 1. The difference between the SPR response at the end of dissociation and that at the beginning of association is denoted as Δ*θ*, which can be used to compare the binding ability of different substances to α‐amylase. The Figure 2d shows the Δ*θ* of the interaction for each flavonoid (200 μM) and acarbose (200 μM) with α‐amylase, respectively.

**Table 1:** Kinetic parameters for interaction of three flavonoids and acarbose with α‐amylase

Flavonoids	*K_a_* (mol^−1^ L s^−1^)	*K_d_* (s^−1^) × 10^–3^	*K_D_* (mM)
Buddleoside	2.5397 ± 0.2	8.2957 ± 0.15	3.2966 ± 0.08^c^
Acacetin	2.7607 ± 006	8.004 ± 0.15	2.924 ± 0.2^c^
Luteolin	4.116 ± 0.1	8.078 ± 0.11	1.9695 ± 0.12^b^
Acarbose	4.7937 ± 0.18	4.5643 ± 0.06	0.933 ± 0.2^a^

Note

Values are mean ± RSD (*n* = 3). Mean values followed by different letters are significantly different (*p* < .05).

As shown in Figure 2 and Table 1, the binding affinity of flavonoids and acarbose for α‐amylase is acarbose > luteolin > acacetin > buddleoside. This result suggests that the binding affinity was affected by the number and position of hydroxyl group (Figure 1). The interaction of these analytes with α‐amylase may be achieved by hydrophobic interactions in nature and then stabilized by hydrogen bonds (Liu et al., 2017; Lu et al., 2017). This is usually enhanced with increasing the number and reactivity of hydroxyl group (Li, Yang, Gao, Zhang, & Wu, 2011; Wang et al., 2010). As shown in Figure 1, the hydroxyl number in acarbose is the largest. However, acarbose is not flavonoids, and the structural differences may have greater effects on the binding ability than that of hydroxyl number. For the three flavonoids, the hydroxylation on positions C′‐4 and C′‐5 of B‐ring remarkably improved the binding ability, resulting in the highest binding affinity of luteolin to α‐amylase (Figure 1) (Al‐Dabbas, Kitahara, Suganuma, Hashimoto, & Tadera, 2006; Cao & Chen, 2012; Lo et al., 2008). Furthermore, the binding affinity of acacetin with α‐amylase (*K_D_*: 2.924 ± 0.2 mM) is greater than that of the buddleoside with α‐amylase (*K_D_*: 3.2966 ± 0.08 mM), indicating that hydroxyl group on position C‐7 of A‐ring is very important for the binding of the flavonoids with α‐amylase. After the hydroxyl group is substituted by a glycoside, steric hindrance may take place, which weakens the binding interaction between buddleoside and α‐amylase (Cao & Chen, 2012; Li et al., 2009). Based on the above results and analysis, it is clearly demonstrated that the SPR sensor may provide more information to evaluate the interaction of the flavonoids with α‐amylase.

### 3.2 Effect of pH and salt on the interaction between three flavonoids and α‐amylase

**Figure 3:**
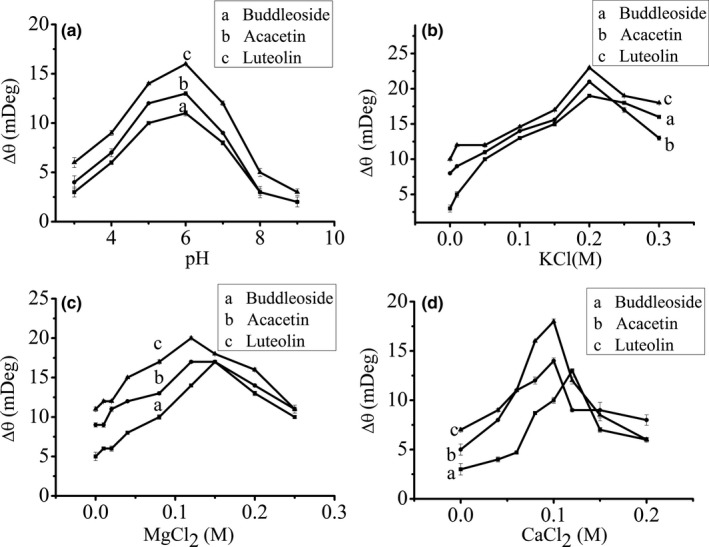
SPR response for the interaction of three flavonoids (200 μM) and α‐amylase (300 μM) with various pH values (a), various concentrations of KCl (b), various concentrations of MgCl_2_ (c), and various concentrations of CaCl_2_ (d) at room temperature

The interaction of between three flavonoids and α‐amylase was studied at different pH using SPR (Figure 3a). At the chosen pH range, the binding affinity of three flavonoids and α‐amylase is strongest at pH 6. This may be ascribed to the α‐amylase isoelectric point (5.04) effect (Liu et al., 2017). When the pH value deviates from the isoelectric point, the associated structure of α‐amylase will change.

SPR results (Figure 3b–d) indicated that KCl, MgCl_2_, and CaCl_2_ play an important role for the interaction between three flavonoids and α‐amylase. The SPR response increases with the increasing of salt concentration in a certain range. The binding ability of three flavonoids to α‐amylase is the strongest at 0.200 M KCl (Figure 3b). It is seen from Figure 3c, the optimal MgCl_2_ concentration is 0.120 M for the binding of acacetin, buddleoside with α‐amylase. However for luteolin, the optimal MgCl_2_ concentration is 0.150 M. Similar results are also observed from Figure 3d, the optimal CaCl_2_ concentration is 0.100 M for the binding of acacetin, buddleoside with α‐amylase. For luteolin, the optimal CaCl_2_ concentration is 0.120 M. The effect of salt concentration on the interaction between three flavonoids and α‐amylase may be due to the following reasons. On the one hand, α‐amylase is a metalloenzyme (Gupta, Gigras, Mohapatra, Goswami, & Chauhan, 2003), and Cl^−^ is activator of the α‐amylase (Kuriki & Imanaka, 1999). It can be seen that the concentration of Cl^−^ is similar at the optimum concentrations for the three salts. The results showed that the concentration of Cl^−^ has significant effect on the binding of enzyme–flavonoids. On the other hand, many studies have confirmed that some metal ions (K^+^, Mg^2+^, and Ca^2+^) can maintain the maximum reactivity of the enzyme (El‐Sayed, Abou‐Dobara, & El‐Fallal, 2017; Kuddus, 2017; Yin et al., 2017). Lower concentration salt may improve the stability of α‐amylase activity. Therefore, the binding affinity of the three flavonoids with α‐amylase can be reinforced by optimal concentration KCl, MgCl_2_, and CaCl_2_. Besides, the enhancement of binding affinity by optimal concentration KCl is the largest, while the enhancements of the binding affinity by optimal concentration MgCl_2_ and CaCl_2_ are similar. This may be because K^+^ has the weakest ionic force, which does not affect the binding of enzyme‐flavonoids. However, further researches are still needed on how ions interfere with the binding of flavonoids and α‐amylase.

### 3.3 Effect of three flavonoids on α‐amylase activity

**Figure 4:**
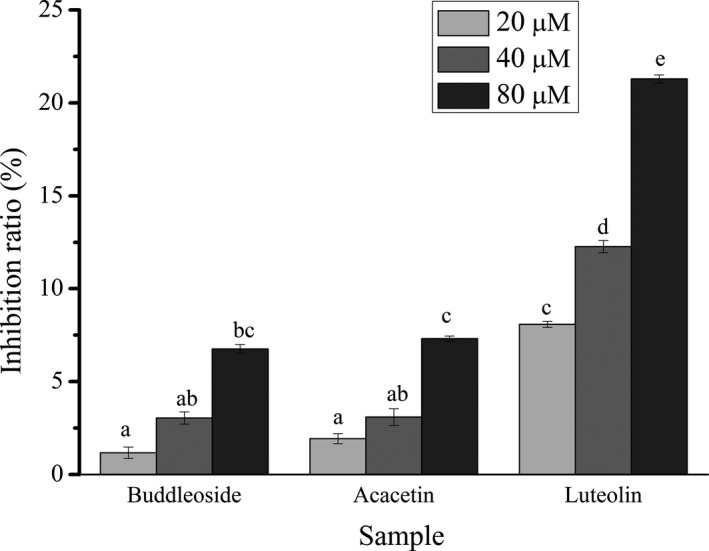
The inhibition effect of buddleoside, acacetin, and luteolin on α‐amylase activity (300 μM). The concentrations of flavonoids were respectively 20, 40, and 80 μM. Results are expressed as the mean ± RSD (*n* = 3). Mean values followed by different letters are significantly different (*p* < .05)

The experimental results in Figure 4 demonstrate that luteolin, acacetin, and buddleoside can dose dependently inhibit α‐amylase activity. At concentration of 80 μM, three flavonoids markedly inhibited α‐amylase activity ranging from 6.76% to 21.29%. The ability of inhibition is luteolin > acacetin > buddleoside, which was in accordance with the results of binding affinity from SPR experiments (Table 1). These results further demonstrate that the free hydroxyl groups in B‐ring and A‐ring (red in Figure 1) are important for the interaction of three flavonoids with α‐amylase. There are several works showed that the hydroxylation on positions C‐3′ and C‐4′ of B‐ring of flavonoids remarkably improved the inhibition for α‐amylase (Lo et al., 2008; Wang et al., 2010). Other scholars demonstrated that flavonoids without one hydroxyl group on any of positions 5, 6, or 7 of A‐ring showed no inhibition for digestive enzyme (Cao & Chen, 2012; Gao, Nishioka, Kawabata, & Kasai, 2004). Besides, hydroxyl group on position C‐7 of A‐ring is very important for the binding of the flavonoids with α‐amylase. The glycosylation of hydroxyl group on flavonoids weakens the binding interaction between flavonoids and α‐amylase (Cao & Chen, 2012; Li et al., 2009).

**Figure 5:**
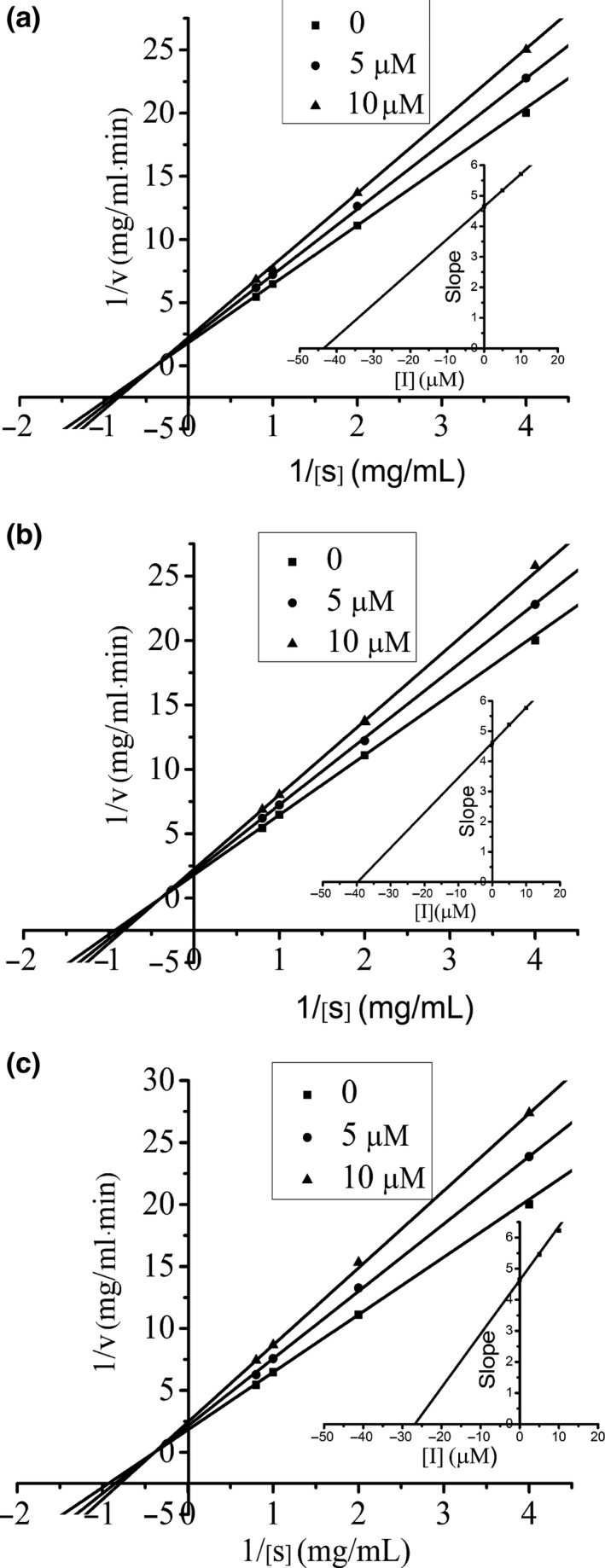
Lineweaver–Burk plot of buddleoside (a), acacetin (b), and luteolin (c) against α‐amylase at different concentrations of starch. The insets ([*I*] vs. the corresponding slope of Lineweaver–Burk plots) were used to calculate the *K_i_* value

The inhibition kinetics of α‐amylase was evaluated by varying the concentration of starch (0.25–1.25 mg/ml) in the absence or presence of three flavonoids. The Figure 5 gives the Lineweaver–Burk plots of three flavonoids. They all have an intersection at the x axis which indicates their inhibitory types are all noncompetitive. *K_i_* values of luteolin, acacetin, and buddleoside are 26.79, 39.73, and 43.55 μM, respectively (Table 2). There are several works showed that the main inhibition mode determined for polyphenolic compounds‐digestive enzymes is noncompetitive (Martinez‐Gonzalez et al., 2017; Yang & Kong, 2016). The result of a noncompetitive inhibition of flavonoid–α‐amylase was supported by the analysis of tea polyphenols‐pancreatic α‐amylase system (Yang & Kong, 2016).

**Table 2:** Inhibitory type and *K_i_* of three flavonoids against α‐amylase

Flavonoids	Inhibitory type	*K_i_* (μM)
Buddleoside	Noncompetitive	43.55
Acacetin	Noncompetitive	39.73
Luteolin	Noncompetitive	26.79

### 3.4 Effect of α‐amylase on antioxidant activity of flavonoids

DPPH radical assay is a rapid, simple, and stable method for the determination of antioxidant capacity of flavonoids. The effect of α‐amylase on antioxidant activity of flavonoids was assessed using DPPH radical assay. Table 3 shows the effect of binding to α‐amylase on the antioxidant activity of three flavonoids (DPPH radical assay). In general, isolated flavonoids have high antioxidant activity. However, the radical scavenging activity of the flavonoids–α‐amylase samples was significantly lower than that of flavonoids alone. It is known that antioxidant activity of phenolic compounds is changed by their binding with fiber and protein. These results reveal that antioxidant activities of three flavonoids are inhibited significantly by binding with α‐amylase (Domínguez Avila, Villegas Ochoa, Alvarez Parrilla, Montalvo González, & González Aguilar, 2018; Jakobek, 2015). The inhibition percentage of antioxidant activity for buddleoside, acacetin, and luteolin are 21.65 ± 0.04%, 26.75 + 0.013%, and 49.93 + 0.037%, respectively (see Table 3), which was in accordance with the results of binding affinity and α‐amylase inhibitory activity (Tables 1 and 2). This result illustrated the antioxidant activity of three flavonoids is closely related to its hydroxyl groups.

**Table 3:** Effect of binding to α‐amylase on the antioxidant activity of three flavonoids (DPPH radical assay)

Flavonoids	Inhibition (%)	Not add α‐amylase IC_50_ (μM)	Add α‐amylase IC_50_ (μM)
Buddleoside	21.65 ± 0.04^a^	65.05 ± 0.02	79.13 ± 0.012
Acacetin	26.75 + 0.013^b^	61.29 + 0.005	77.76 + 0.004
Luteolin	49.93 + 0.037^c^	50.14 + 0.014	75.16 + 0.013

Note

Inhibition (%) = (add α‐amylase IC_50_ − not add α‐amylase IC_50_)/not add α‐amylase IC_50_.

Values are mean ± RSD (*n* = 3). Mean values followed by different letters are significantly different (*p* < .05).

## 4 CONCLUSION

To conclude, a low‐cost, simple, sensitive, and label‐free method was successfully applied to investigate real‐time interactions of the luteolin, acacetin, and buddleoside with α‐amylase, and the influence of external factors (pH, KCl, MgCl_2_, and CaCl_2_) on the interaction. The affinity order is luteolin > acacetin > buddleoside. In addition, the binding of three flavonoids with α‐amylase can not only inhibit α‐amylase activity with noncompetitive mode, but also decrease the antioxidant activities of three flavonoids. Furthermore, the results reveal the significance of hydroxyl on C′‐4, C′‐5 of B‐ring, and C‐7 of A‐ring of three flavonoids for binding with α‐amylase. Besides, the glycosylation of hydroxyl group on flavonoids weakened the binding interaction between flavonoids and α‐amylase. These results provide scientific support for the proper use of luteolin, acacetin, and buddleoside as potential inhibitors of the α‐amylase.

## CONFLICT OF INTEREST

The authors notify that there are no conflicts of interest.

## ETHICAL APPROVAL

This study does not involve any human or animal testing.
